# Role of gut microbiota in the modulation of atherosclerosis-associated immune response

**DOI:** 10.3389/fmicb.2015.00671

**Published:** 2015-06-30

**Authors:** Dmitry A. Chistiakov, Yuri V. Bobryshev, Emil Kozarov, Igor A. Sobenin, Alexander N. Orekhov

**Affiliations:** ^1^Department of Molecular Genetic Diagnostics and Cell Biology, Division of Laboratory Medicine, Institute of Pediatrics, Research Center for Children’s Health, MoscowRussia; ^2^The Mount Sinai Community Clinical Oncology Program, Mount Sinai Comprehensive Cancer Center, Mount Sinai Medical Center, Miami Beach, FLUSA; ^3^Laboratory of Angiopathology, Institute of General Pathology and Pathophysiology, Russian Academy of Sciences, MoscowRussia; ^4^Faculty of Medicine, School of Medical Sciences, University of New South Wales, Sydney, NSWAustralia; ^5^School of Medicine, University of Western Sydney, Campbelltown, NSWAustralia; ^6^Department of Oral and Diagnostic Sciences, Columbia University, New York, NYUSA; ^7^Laboratory of Medical Genetics, Russian Cardiology Research and Production Complex, MoscowRussia; ^8^Institute for Atherosclerosis, Skolkovo Innovation Center, MoscowRussia; ^9^Department of Biophysics, Biological Faculty, Moscow State University, MoscowRussia

**Keywords:** intestinal microbiota, immune system, atherosclerosis, atherogenesis, arteries

## Abstract

Inflammation and metabolic abnormalities are linked to each other. At present, pathogenic inflammatory response was recognized as a major player in metabolic diseases. In humans, intestinal microflora could significantly influence the development of metabolic diseases including atherosclerosis. Commensal bacteria were shown to activate inflammatory pathways through altering lipid metabolism in adipocytes, macrophages, and vascular cells, inducing insulin resistance, and producing trimethylamine-*N*-oxide. However, gut microbiota could also play the atheroprotective role associated with anthocyanin metabolism and administration of probiotics and their components. Here, we review the mechanisms by which the gut microbiota may influence atherogenesis.

## Introduction

Intestinal microorganisms are crucially involved in the induction and modulation of mucosal tolerance (Noverr and Huffnagle, 2004; Sanz and De Palma, 2009; Peterson and Cardona, 2010; Tlaskalová-Hogenová et al., 2011; Huang et al., 2013; Ma et al., 2015). Alterations in mucosal tolerance induced by imbalanced gut microflora may lead to acute or chronic inflammation (Sanz and De Palma, 2009; Chow et al., 2010; Lee and Mazmanian, 2010; Jarchum and Pamer, 2011; Swiatczak and Rescigno, 2012; Magrone and Jirillo, 2013; Chistiakov et al., 2015). The effects of imbalanced microbiota are not restricted by gastrointestinal abnormalities but could have systemic impact on immunity (Noverr and Huffnagle, 2004; Huang et al., 2013; Ma et al., 2015). The gut microbiota appears to play role in atherosclerosis, a chronic vascular inflammatory disease, through several mechanisms. In this review, we consider the role of intestinal microbiota in modulation of atherosclerotic inflammatory response.

## Gut Microbiota and Atherogenesis

### Impact of Intestinal Microflora to Metabolic Abnormalities, a Risk Factor for Atherosclerosis

Recent data demonstrate that intestinal microorganisms could influence lipid metabolism and act as environmental factors triggering development of metabolic and cardiovascular diseases (Vrieze et al., 2010; Goldsmith and Sartor, 2014). The lack of gut microbiota in germ-free apolipoprotein E (ApoE)-null mice, an experimental model of human atherosclerosis, was found to induce the development of atherosclerotic plaques even when animals were fed a standard low-cholesterol diet. Colonization with normal human microbiota prevented atherogenesis in germ-free ApoE-null mice fed a standard low-cholesterol diet but not a diet with high cholesterol content (Stepankova et al., 2010). Indeed, these observations suggest on the atheroprotective effects of human colonic commensal bacteria.

Increased intestinal microbiota-derived lipopolysaccharide (LPS) load from the colon lumen was shown to be associated with various metabolic abnormalities including induction of adipose inflammation and insulin resistance (Cani et al., 2007). Bacterial LPS could be delivered from the gut to the circulation through chylomicron-associated transport and *via* tight junctions in the epithelial lining (Caesar et al., 2010). LPS is absorbed by enterocytes and transferred to the Golgi apparatus where chylomicrons synthesized by enterocytes are stored before secretion (Sabesin and Frase, 1977). Inhibition of chylomicron formation suppressed intestinal LPS absorption (Ghoshal et al., 2009). High-fat meal intake increases circulating levels of LPS (Amar et al., 2008).

Enhanced LPS load across the tight junctions of the gut epithelium was observed in animal models of human obesity and associated with the rearrangement of tight junction proteins, reduced epithelial barrier function, and increased gut permeability, endotoxemia, and inflammation (Brun et al., 2007; Cani et al., 2008). Administration of antibiotics or prebiotic oligofructose was shown to improve the integrity of intestinal epithelium and decrease serum low density lipoproteins (LDLs) and liver inflammation (Cani et al., 2009). Similarly, treatment of ApoE-null mice with a mixture of eight probiotics VSL#3 had anti-inflammatory effects on the gastrointestinal tract by decreasing aortic atherosclerosis, steatohepatitis, and low-grade inflammation of intestinal and mesenteric adipose tissues induced by a high-fat cholesterol intake (Mencarelli et al., 2012). Therefore, high-fat food leads to unfavorable changes in gut microbiota that contributes to induction of metabolic abnormalities in the host organism associated with primary intestinal epithelial dysfunction and induction of gastrointestinal inflammation that could be reversed by administration of probiotics and their products (Lee, 2013). It is essential to note here that ApoE mice naturally develop atherosclerotic plaque even in the absence of high fat diet (HFD); however, HFD accelerates this process (Imaizumi, 2011).

### Gut Microbiota and Phosphatidylcholine Metabolism

Recently, a proatherogenic role of the gut microbiota in the metabolism of phosphatidylcholine was shown (Wang et al., 2011b). Intestinal microbiota metabolizes choline and phosphatidylcholine to trimethylamine (TMA), which is further converted to a proatherogenic compound, trimethylamine-*N*-oxide (TMAO; Koeth et al., 2013). Dietary L-carnitine, a TMA abundant in red meat, is metabolized by intestinal microbiota to TMAO and accelerates atherosclerosis in ApoE-null mice through changes in microbial composition and increased colon production of TMA and TMAO (Ferguson, 2013). The production of TMAO was dependent on variability of the gut microbiota species. Gut bacteria such as *Prevotella* were found to produce more TMAO than *Bacteroides.* Indeed, omnivorous people produced more TMAO than did vegetarians. Increased plasma L-carnitine levels were detected in patients with cardiovascular abnormalities and were associated with increased cardiovascular risk (Koeth et al., 2013). Functional studies showed that TMAO inhibited reverse cholesterol transport (RCT) and promoted accumulation of cholesterol in macrophages through increasing cell surface expression of proatherogenic scavenger receptors (SRs) CD36 and SRA (Wang et al., 2011b), reducing synthesis of bile acids from cholesterol, and decreasing expression of bile acid transporters in the liver (Koeth et al., 2013). Indeed, increased meat consumption could elevate the dose of L-carnitine and enhance TMAO production in the gut thereby contributing to higher cardiovascular risk. These findings create essential prerequisites for possibility to mediate the atherosclerotic risk through dietary or pharmacological manipulation of the gut microbiota. However, due to the complexity of the interactions among host genetics, host diet, and microbiota and still-limited understanding of specific mechanisms of L-carnitine effects on atherogenesis, it is too early to suggest whether this should be translated into specific dietary recommendations.

### Reverse Cholesterol Transport

Gut microorganisms were found to be involved in the regulation of food fat and cholesterol uptake by enterocytes through several pathways including production of bioactive short-chain fatty acids such as butyrate and acetate, LPS-mediated activation of Toll-like receptor 4 (TLR4), and glucagon-like peptide-2-dependent regulation of epithelial integrity (Musso et al., 2010; Lee-Rueckert et al., 2013).

Atherosclerosis was shown to be a frequent consequence of hypercholesterolemia leading to the accumulation of cholesterol in the vascular wall. RCT is a mechanism that counteracts the deposition of excess cholesterol in peripheral tissues. RCT-mediated eﬄux of cholesterol from foam cells accumulated in the intra-intimal regions of atherosclerotic vessels is believed to be atheroprotective in early atherosclerotic stages (Tall et al., 2008). The systemic cholesterol balance and metabolism are regulated by liver X receptors (LXRs) and nuclear receptors responsive to stimulation by oxysterols (Zelcer and Tontonoz, 2006). LXR is critical for cholesterol homeostasis controlling cholesterol levels by inducing RCT and promoting degradation of lipid metabolism-related receptors such as low density lipoprotein (LDL) receptor, very low density lipoprotein (VLDL) receptor, and adiponectin receptor 2 (AdipoR2) through transcriptional induction of Idol (inducible degrader of the LDLR; Zelcer et al., 2009; Hong et al., 2010). In dividing T cells, LXR suppresses proliferation through activation of Sterol Regulatory Element (SRE)-binding protein (SREBP)-mediated cholesterol synthesis and induction of the oxysterol-metabolizing enzyme SULT2B1 (sulfotransferase 2B1) associated with inactivation of the sterol ATP-binding cassette transporter ABCG1 that uncouples cholesterol transport (Bensinger et al., 2008).

Liver X receptors agonists regulate RCT in macrophages, liver, and small intestine. In macrophages, LXR stimulates expression of sterol transporters ABCA1 and ABCG1 responsible for cholesterol eﬄux (Tall et al., 2008). In the liver, LXR up-regulates expression of cholesterol-7-α-hydroxylase (CYP7A1) that is involved in cholesterol catabolism to bile acids (Chiang et al., 2001). In macrophages, TLR2 and TLR4 suppress stimulation of LXRs through both myeloid differentiation primary response gene (MyD)88-dependent and independent pathways while apolipoprotein A1, an essential protein component of high density lipoprotein (HDL) particles and TLR2, TLR4, and CD14 agonist, utilizes MyD88-dependent mechanism to induce RCT (Smoak et al., 2010). In summary, LXRs play a remarkable role on crossroads of metabolic, cell cycle, and immune signaling.

### Gut Microbiota and Anthocyanin Metabolism

Recent findings showed that the intestinal microbiota has a new mechanism associated with the metabolism of anthocyanin that could be useful for atheroprotection. Protocatechuic acid (PCA), a metabolite of cyanidin-3-O-β-glucoside (α-G) was shown to have a profound anti-atherogenic effect (Wang et al., 2010). PCA was found to promote cholesterol eﬄux from macrophages through activation of expression of ABCA1 and ABCG1 by down-regulating microRNA (miR)-10b that target both cholesterol transporters (Hazen and Smith, 2012; Wang et al., 2012a). PCA is directly produced by gut microbiota from Cy-3-G. The main fruit sources of Cy-3-G and other cyanidins are blackberries and bilberries. Other dietary sources include chokeberries, boysenberries, elderberries, purple vegetables (such as carrots and yams), black raspberries, and *Hibiscus sabdariffa* extract. In ApoE-null mice, both PCA and Cy-3-G were reported to attenuate atherosclerosis (Wang et al., 2011a). Cy-3-G was able to improve serum cholesterol levels in ApoE-null mice and increase formation of bile acids through activation of liver expression of CYP7A1 *via* direct binding to LXRα (Wang et al., 2012b). In addition, Cy-3-G could efficiently block atherosclerotic progression in ApoE-null mice fed on a high-fat diet by improving hypercholesterolemia-induced endothelial dysfunction through reducing circulating levels of cholesterol and 7-ketocholesterol and restoring production of nitric oxide (NO) by vascular endothelial cells (Zhang et al., 2013). However, compared to Cy-3-G, PCA showed the anti-atherogenic effect at physiologically reachable concentrations that makes it to be potentially significant for therapeutic use.

In intimal macrophages and vascular smooth muscle cells (VSMCs), TLR2/4/MyD88 pathway is involved in support of the proatherogenic intracellular cholesterol accumulation stimulated by bacterial LPS derived from *E. coli* and oxLDL (Higashimori et al., 2011). In foam cells, LXRs and TLR2/4 were shown to regulate intracellular cholesterol transport by reciprocal inhibition of each other (Cao et al., 2007; Chen et al., 2008). The proinflammatory anthocyanin pigment Cy-3-G presented in the human diet was shown to inhibit TLR4-mediated proinflammatory signaling in macrophages by up-regulation of the LXRα/ABCG1 axis that activates RCT resulted in subsequent disrupting lipid rafts by depleting cholesterol and limiting translocation of TLR4 to lipid rafts (Fu et al., 2014).

### Atheroprotective Role of Probiotic Microorganisms

Consumption of a probiotic strain DSM 9843 of *Lactobacillus plantarum* by men with carotid atherosclerosis showed some beneficial effects for the host associated with increase of the bacterial diversity in the gut and with changes in colon levels of certain short-chain fatty acids (Karlsson et al., 2010). Similarly, Naruszewicz et al. (2002) demonstrated that another strain 299v of *L. plantarum* was able to reduce several cardiovascular disease risk factors in smokers including positive metabolic changes, decrease in levels of proinflammatory cytokine IL-6, and reduced adhesion of monocytes to endothelial cells. On the other hand, administration of *L. delbrüeckii* in ApoE-null mice fed on a hypercholesterolemic diet had only modest atheroprotective effect (Portugal et al., 2006). Limited anti-atherogenic effects of human intestinal microbiota in case of uptake of a high-fat diet may be explained by positive association of some human commensals such as *Firmicutes* and *Bacteroidetes* with obesity due to increased capability of these microbes to metabolize fiber into short chain fatty acids that could be converted to fat in high lipid load (Turnbaugh et al., 2006). In mice fed a high-fat diet, consumption of probiotic bacteria *L. rhamnosus* GG and *L. sakei* NR28 had beneficial anti-obesity effects through reduction in the small intestine frequency of obesity-associated commensals *Firmicutes* and *Bacteroidetes*, decrease of epididymal fat mass and down-regulation of liver lipid-synthesizing enzymes (Ji et al., 2012).

### Impact of Gut Microbiota to Metabolic Inflammation and Atherosclerosis

Accumulated data have revealed a close relationship between inflammatory and metabolic pathways. Indeed, inflammation was recognized to significantly contribute to the pathogenesis of obesity, insulin resistance, and atherosclerosis. Traditionally, colonic bacteria were considered as agents activating inflammatory mechanisms. This is supported by multiple data showing the link between the gut microbiota, inflammation, and autoimmunity.

Colonic microbiota could stimulate infiltration of macrophages in the adipose tissue by providing inflammatory stimuli such as LPS and enhancing energy intake from the food that leads to adipocyte hypertrophy (Bäckhed et al., 2004). Free fatty acids and bacterial LPS act synergistically in stimulation of adipose inflammation. Therefore, it is difficult to determine specific contribution of the gut microbiota to metabolic inflammation.

Toll-like receptor 4 seems to play a role of the molecular link between metabolism, nutrition, and inflammation. Proinflammatory macrophages infiltrating the adipose tissue are activated by fatty acids through TLR2 and TLR4 stimulation (Nguyen et al., 2007). Adipocyte-derived saturated fatty acids also induce nuclear factor (NF)-κB-mediated expression of tumor necrosis factor (TNF)-α and other proinflammatory genes *via* TLR4-dependent mechanism (Suganami et al., 2007). A paracrine loop involving fatty acids and TNF-α mediates reciprocal proinflammatory changes in adipocytes and infiltrating macrophages during the adipose-associated inflammation (Suganami et al., 2005).

In fact, LPS represents an endotoxin whose production by gut microbes could lead to chronic low-grade inflammation and contribute to progression of obesity, insulin resistance, metabolic syndrome, and diabetes, e.g., well recognized atherogenic risk factors (Cani et al., 2007; Jialal and Rajamani, 2014). Increased intestinal permeability results in elevated endotoxin levels in the circulation that could affect lipid metabolism and induce low-grade inflammatory response associated with higher cardiovascular risk (Teixeira et al., 2012). Unlike high-dose LPS, low-dose LPS does not initiate marked activation of NF-κB, mitogen-activated protein kinases (MAPK), phosphatidylinositol-3-kinases (PI3K), or anti-inflammatory mediators. Instead, low-dose LPS induce hepatocyte nuclear factor 1 homeobox B (HNF1B) through Toll-interacting protein-mediated generation of mitochondrial reactive oxygen species, allowing mild induction of proinflammatory mediators. Low-dose LPS also down-regulates PI3K and related negative regulators of inflammatory genes (Maitra et al., 2012). In addition to the modest activation of proinflammatory genes, low-dose endotoxin was found to reduce expression of proteins involved in reverse cholesterol transport such as ABCA1/ABCG1 and SR-B1 in murine macrophages (Maitra and Li, 2013). Even superlow doses of LPS could show deleterious effects in primary cultures of mouse macrophages by inducing mitochondrial fission and cell necroptosis through ubiquitination and degradation of mitofusin 1 (Mfn1; a molecule needed for proper mitochondrial fusion) and dephosphorylation and activation of Drp1 (a molecule responsible for mitochondrial fission and cell necroptosis). This process is mediated by interleukin 1 receptor-associated kinase (IRAK-1) and receptor-interacting protein 3 kinase (RIP3), e.g., molecules critical for the assembly of the necrosome complex (Baker et al., 2014). Indeed, LPS at low dose and superlow dose exploit different pathogenic mechanisms in macrophage cytotoxicity.

In addition to LPS, the microbiota produces many other proinflammatory molecules including flagellin, peptidoglycan, etc. recognized by TLRs and other PRRs. For example, peptidoglycan derived from the gut microbiota systemically drives the innate immune system stimulating killing two major pathogens *Streptococcus pneumoniae* and *Staphylococcus aureus* by bone marrow neutrophils (Clarke et al., 2010). Peptidoglycan-mediated mechanism of neutrophil activation requires recognition by the PRRs such as the nucleotide-binding, oligomerization domain-containing protein-1 (Nod1) and TLR4 (Chamaillard et al., 2003).

In human atherosclerotic lesions, several TLRs including TLR2 and TLR4 are expressed in proinflammatory macrophages and vascular cells (Monaco et al., 2009). Activation of TLR2 and TLR4 is mediated by MyD88 that in turn is responsible for providing proatherogenic and proinflammatory signals (Fitzgerald et al., 2001). The atherogenic role of these signaling molecules is supported by observations in ApoE-null mice deficient for TLR2, TLR4, or MyD88 that have attenuated atherosclerosis (Michelsen et al., 2004). In MyD88-null mice, cholesterol metabolism and uptake of oxidized LDL (oxLDL) were not altered but macrophage adhesion to the vascular endothelium was impaired due to reduced expression of chemokines (Björkbacka et al., 2004).

Bacterial LPS were shown to activate proinflammatory production of matrix metalloproteinase (MMP)-9 through TLR2/4 activation in macrophages, VSMCs, and endothelial cells that could lead to the atherosclerosis-associated vascular remodeling and plaque destabilization (Li et al., 2012; Paolillo et al., 2012). LXRα could efficiently block up-regulation of MMP-9 in LPS-stimulated macrophages by repressing TLR2/4-dependent stimulation (Castrillo et al., 2003). Indeed, a crosstalk between proatherogenic TLR2/4 and atheroprotective LXR signaling in vascular cells and macrophages could contribute to the development of atherosclerotic disease and mediate atherogenic or anti-atherogenic effects of bacterial components such as LPS and bacterial metabolites such as Cy-3-G.

## Concluding Remarks

**FIGURE 1:**
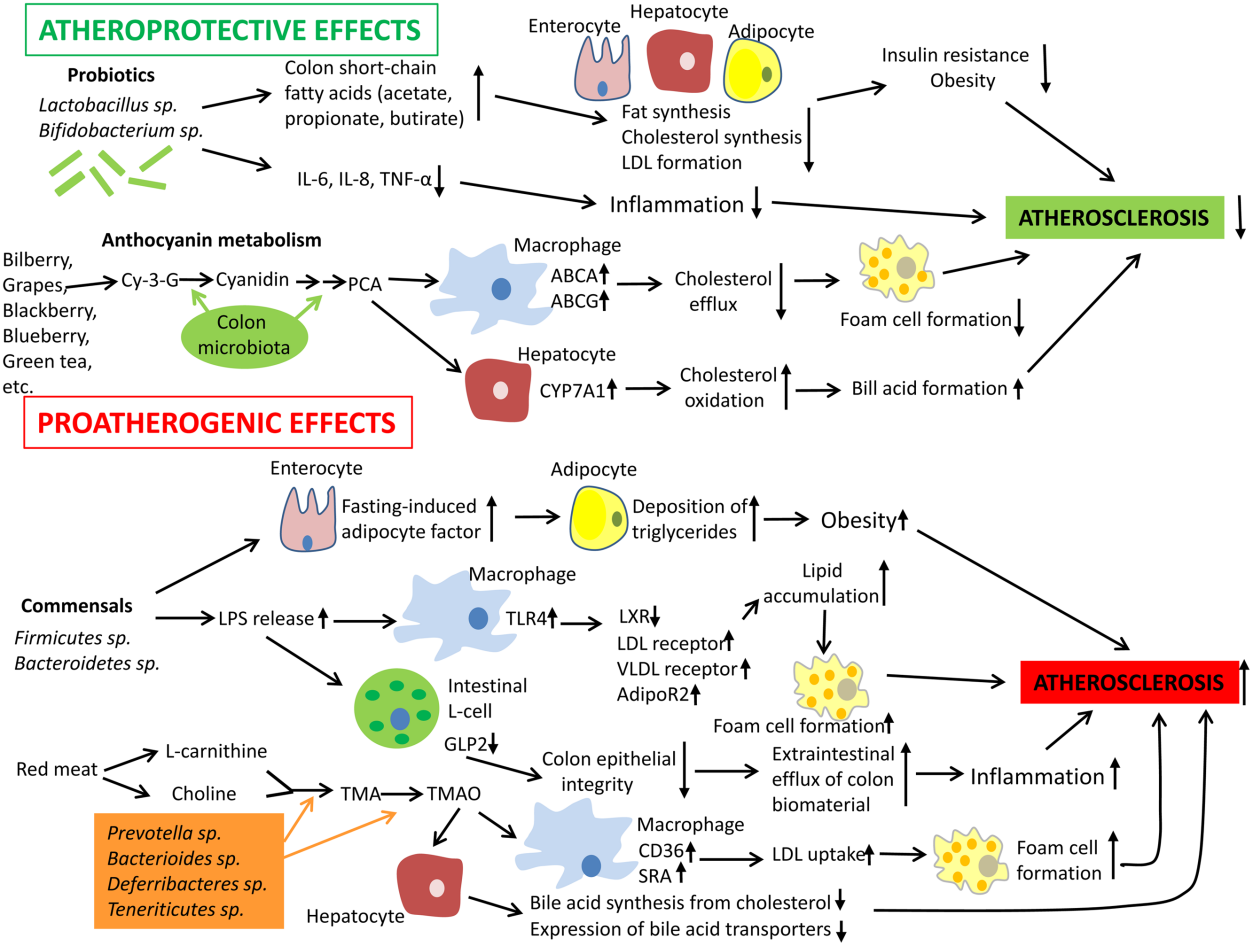
**Mechanisms by which gut microbiota could influence the development of atherosclerosis**. Intestinal microbiota may have both pro- and anti-atherogenic effects. Probiotics have the atheroprotective activity through the release of bioactive short-chain fatty acids that in turn inhibit fat synthesis in adipocytes and enterocytes and suppress cholesterol biosynthesis and formation of proatherogenic low density lipoproteins (LDL) in the liver. In addition, probiotics and their products could down-regulate inflammation thereby indirectly decreasing the atherosclerosis progression. In addition, anthocyanins presented in some barriers, fruits, and green tea could be metabolized by colon microflora to protocatechuic acid (PCA), a bioactive molecule that could increase expression of ATP-binding cassette transporters ABCA and ABCG and in macrophages and therefore stimulate cholesterol eﬄux and inhibit macrophage transformation to foam cells, a hallmark of early atherosclerosis. PCA could stimulate cholesterol catabolism and bile acid synthesis *via* up-regulation of liver cholesterol 7 α-hydroxylase CYP7A1, an enzyme, which oxidizes cholesterol. However, gut microbiota could also exhibit proatherogenic effects. Commensal bacteria such as *Firmicutes* sp. and *Bacteroidetes* sp. release lipopolysaccharides (LPS) that could be recognized by Toll-like receptor (TLR)4 and down-regulate expression of transcription factor liver X receptor (LXR)-á (suppressor of cholesterol uptake) while expression of proatherogenic LDL receptor, very low density lipoprotein (VLDL) receptor, and adiponectin receptor 2 (AdipoR2) become up-regulated in macrophages. Indeed, this leads to elevated lipid uptake by macrophages promoting their transformation to foam cells. Furthermore, intestinal-derived LPS were shown to decrease the production of glucagon-like peptide 2 (GLP2) intestinal neuroendocrine L-cells, which results in weakened colon epithelial integrity, increased eﬄux of colon biomaterial outside the intestine, and enhanced inflammation. Finally, some species of intestinal microbiota were found to show increased capacity to metabolize choline and L-carnitine, which are enriched in red meat, to trimethylamine (TMA) and trimethylamine-*N*-oxide (TMAO). Elevated levels of TMAO are associated with increased cardiovascular risk. TMAO is able to activate expression of LDL scavenger receptors SRA and CD36 in macrophages thereby stimulating LDL uptake and formation of foam cells. In hepatocytes, TMAO suppress both bile acid synthesis from cholesterol and expression of bile acid transporters. Abbreviations: Cy-3-G, cyanidin-3-O-â-glucoside; IL-6, interleukin 6; TNF-á, tumor necrosis factor á; SRA, scavenger receptor A.

The mechanisms responsible for the induction of immune tolerance in atherogenesis have been discussed in a number of recent reviews (Ketelhuth and Hansson, 2011; Perrins and Bobryshev, 2011; Broder et al., 2013; Van Brussel et al., 2014). Mechanisms by which gut microbiota could influence the development of atherosclerosis are summarized in **Figure 1**. Albeit the information about the impact of the intestinal microbiota to atherogenesis is still limited and ‘fragmented,’ accumulating knowledge unambiguously indicates that the intestinal microbiota influences host immunity and contributes to atherosclerosis.

## Conflict of Interest Statement

The authors declare that the research was conducted in the absence of any commercial or financial relationships that could be construed as a potential conflict of interest.
